# “Terrible Triad” Injury in an Adolescent Patient With a High-Energy Femoral Shaft Fracture: A Case Report

**DOI:** 10.7759/cureus.80339

**Published:** 2025-03-10

**Authors:** Alexandros E Koskiniotis, Nikolaos Stefanou, Efstathios Konstantinou, Efstratios D Athanaselis, Sokratis Varitimidis

**Affiliations:** 1 Department of Orthopaedic Surgery and Musculoskeletal Trauma, University Hospital of Larissa, Larissa, GRC

**Keywords:** fatigue of osteosynthesis material, femur fractures, intramedullary femur nailing, lower extremity trauma, osteosynthesis, patellar tendon rupture, refracture, s: fat embolism syndrome(fes)

## Abstract

Although femoral shaft fractures are typically treated with intramedullary nailing, alternative fixation methods like plating are required in select cases, particularly when nailing may be contraindicated due to respiratory complications or other patient-specific factors. Currently, there are no established guidelines for the optimal surgical fixation method of femoral shaft fractures in polytrauma patients with respiratory dysfunction. We present the case of a young patient with a diaphyseal femur fracture who underwent multiple surgical interventions within three months due to recurrent injuries and complications, primarily resulting from non-adherence to postoperative guidelines. We describe this sequence as a "terrible triad injury", consisting of primary fixation of the femoral fracture, revision of the first osteosynthesis due to recurrent trauma and implant failure, and a subsequent patellar tendon rupture.

## Introduction

Femoral shaft fractures are common orthopedic injuries with a bimodal age distribution. They often occur in younger patients due to high-energy trauma, which may be associated with life-threatening injuries (e.g., pulmonary or cerebral), or in older patients due to low-energy mechanisms, such as simple falls [1]. Intramedullary nailing is the gold standard treatment for these fractures, providing excellent functional outcomes and advantages such as early weight-bearing and minimal soft tissue damage, thereby reducing surgical site complications [2]. However, complications like respiratory dysfunction due to fat embolism may contraindicate nailing, making open reduction and internal fixation (ORIF) with a plate a more suitable alternative [3]. This case report presents a young patient with a femoral shaft fracture who required multiple surgical interventions within three months due to consecutive injuries and complications, primarily resulting from non-adherence to postoperative guidelines. We describe this sequence as a "terrible triad injury", comprising primary fixation of the femoral fracture, revision of the first osteosynthesis due to recurrent trauma and implant failure, and a subsequent patellar tendon rupture.

## Case presentation

**Figure 1:**
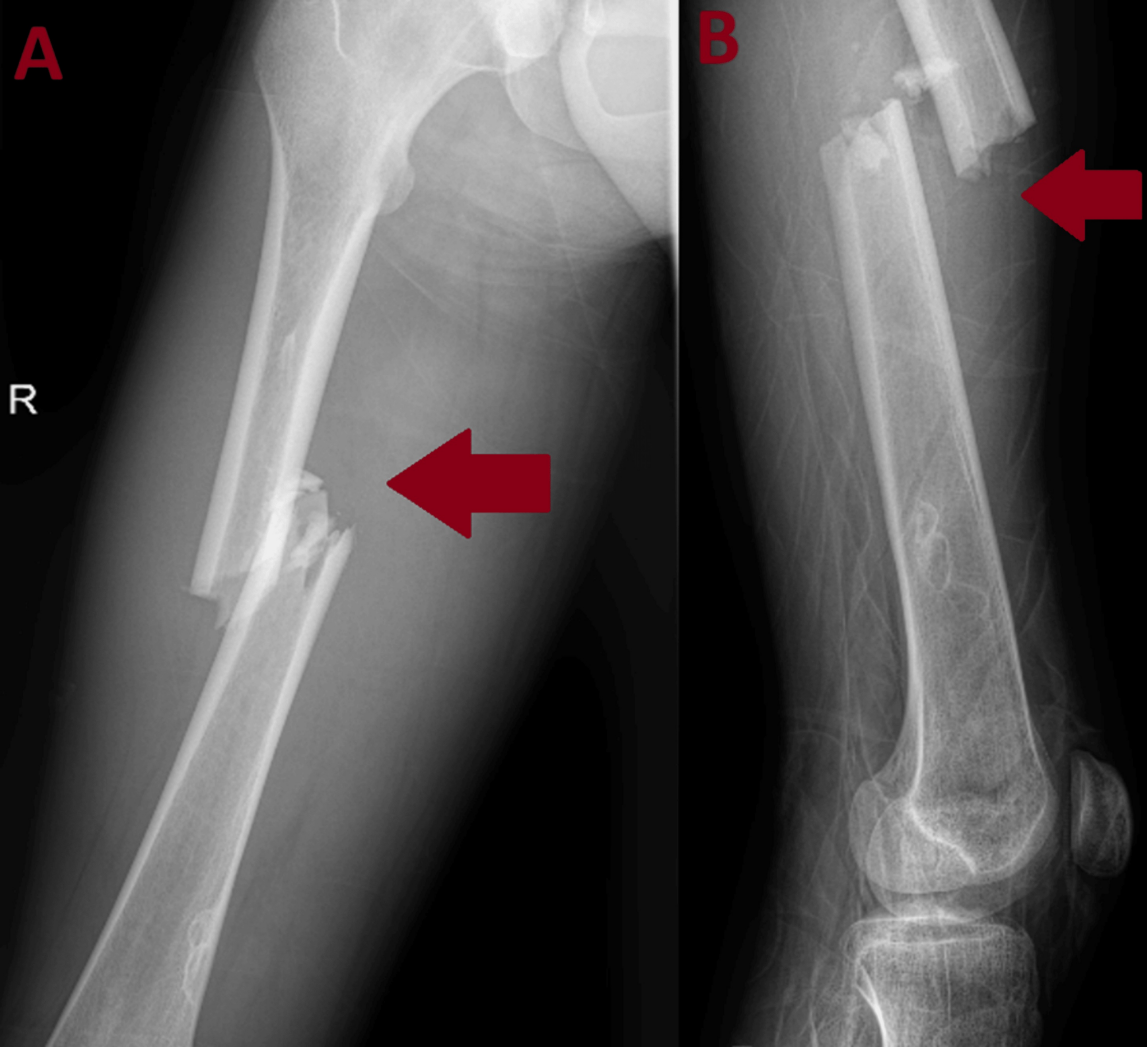
Preoperative images of the right femoral shaft fracture A) Anteroposterior X-ray of the femur. B) Lateral X-ray of the femur

A 17-year-old male with no significant medical history presented to our emergency department following a motorcycle accident. He had sustained a diaphyseal fracture of the right femur without significant soft tissue trauma and an ipsilateral sixth rib fracture (Figure 1).

**Figure 2:**
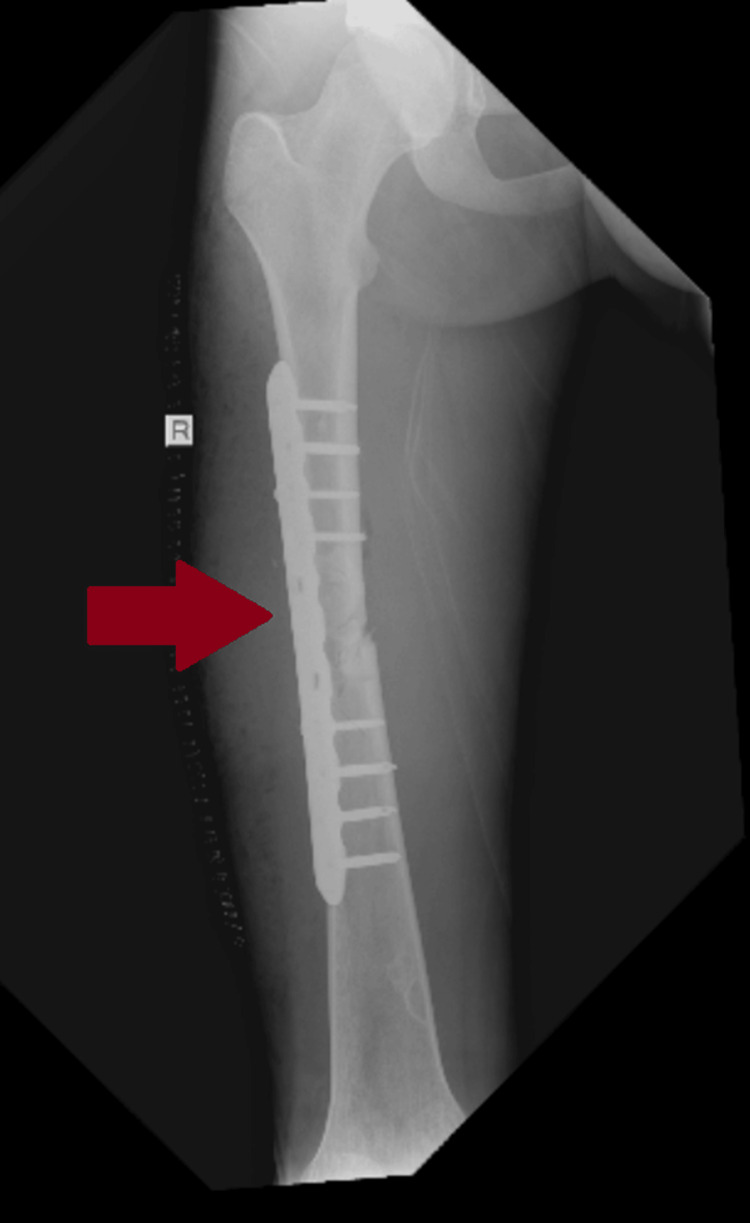
Immediate anteroposterior postoperative X-ray after fixation of the diaphyseal femur fracture with a plate

Despite being hemodynamically stable, the patient reported shortness of breath and chest pain unrelated to the rib fracture. His oxygen saturation (SpO₂) was 90%, accompanied by tachypnea. A clinical evaluation by a pulmonologist and a chest CT scan revealed no specific findings; however, a low-grade fat embolism was strongly suspected. Given this diagnosis, the use of reamed intramedullary nailing was avoided due to concerns about exacerbating respiratory symptoms. Instead, ORIF with a plate was performed to minimize operative time and potential complications (Figure 2).

Postoperatively, the patient was instructed to perform toe-touch weight-bearing and active and passive range-of-motion exercises. Three days after surgery, his respiratory condition improved, and he was discharged from the hospital.

**Figure 3:**
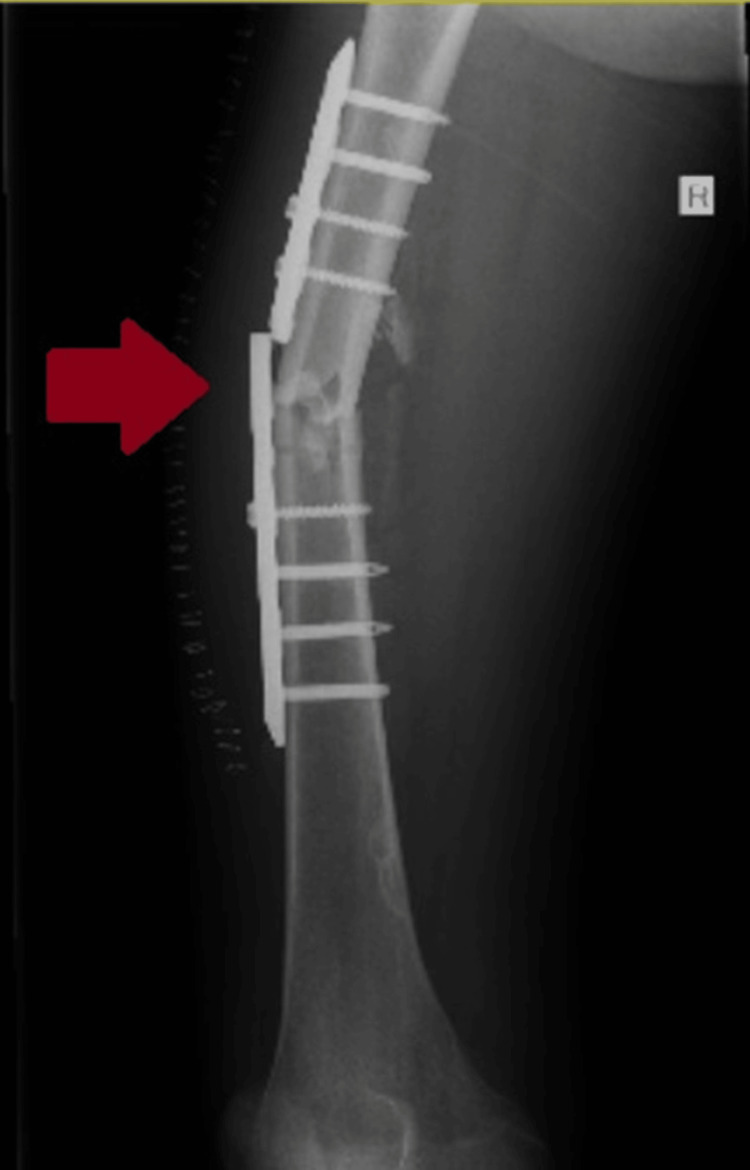
Failure of the osteosynthesis due to breakage of the plate after a second accident, resulting in a re-fracture at the operated site

Unfortunately, the patient sustained another fall 19 days later while bathing unsupervised. This resulted in plate failure and a re-fracture at the original femoral diaphysis site (Figure 3).

**Figure 4:**
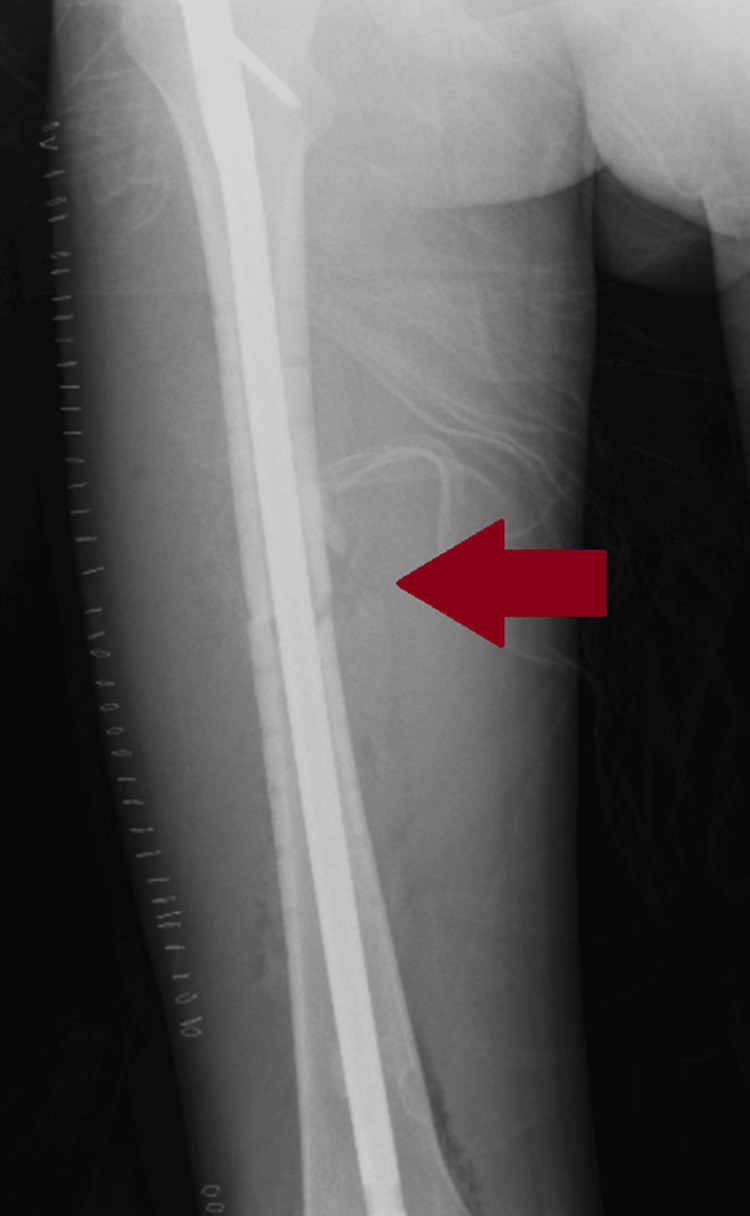
Removal of the plate and intramedullary nail fixation of the femoral shaft re-fracture

Since his respiratory symptoms had resolved, the fracture was treated with an intramedullary nail during a second operation (Figure 4).

The patient was instructed on strict weight-bearing restrictions under supervision and prescribed a rehabilitation program. At his two-month follow-up, he demonstrated significant improvement, with a near-normal range of motion and the ability to walk unaided.

**Figure 5:**
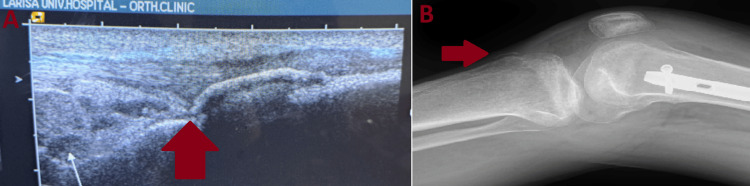
Ultrasound and X-ray imaging of the injured knee after the third traumatic incident of the patient A bony avulsion can be seen in both images A) Ultrasound image of the injured knee in longitudinal view. A partial rupture of the patellar tendon and a bone avulsion can be seen near the tibial tubercle. B) Lateral X-ray of the injured knee

However, non-compliance with postoperative instructions once again led to complications. After growing over-confident due to his excellent recovery, the patient resumed motorbike riding, against medical advice, and sustained a low-velocity fall. This time, he ruptured the patellar tendon in his previously operated limb, though no new fractures were identified (Figure 5).

**Figure 6:**
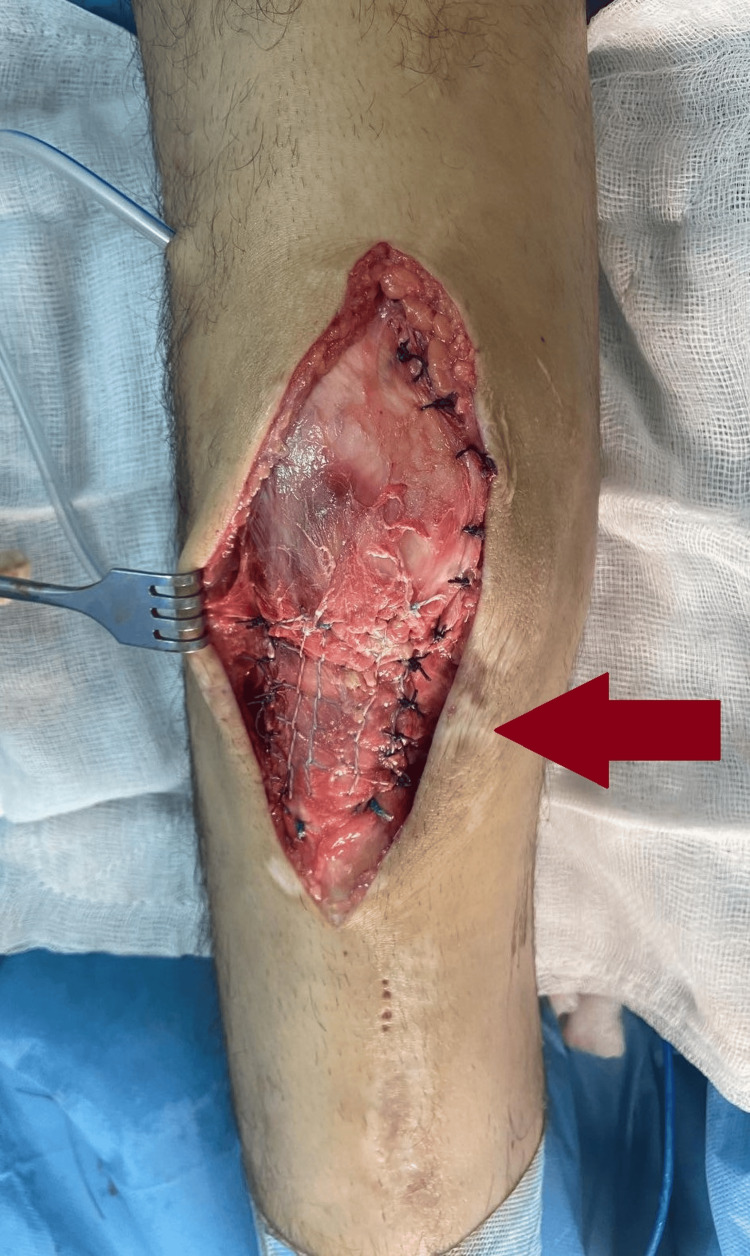
Intraoperative image of surgical repair of the ruptured patellar tendon

Surgical repair of the ruptured tendon was performed, marking his third operation within three months (Figure 6).

**Figure 7:**
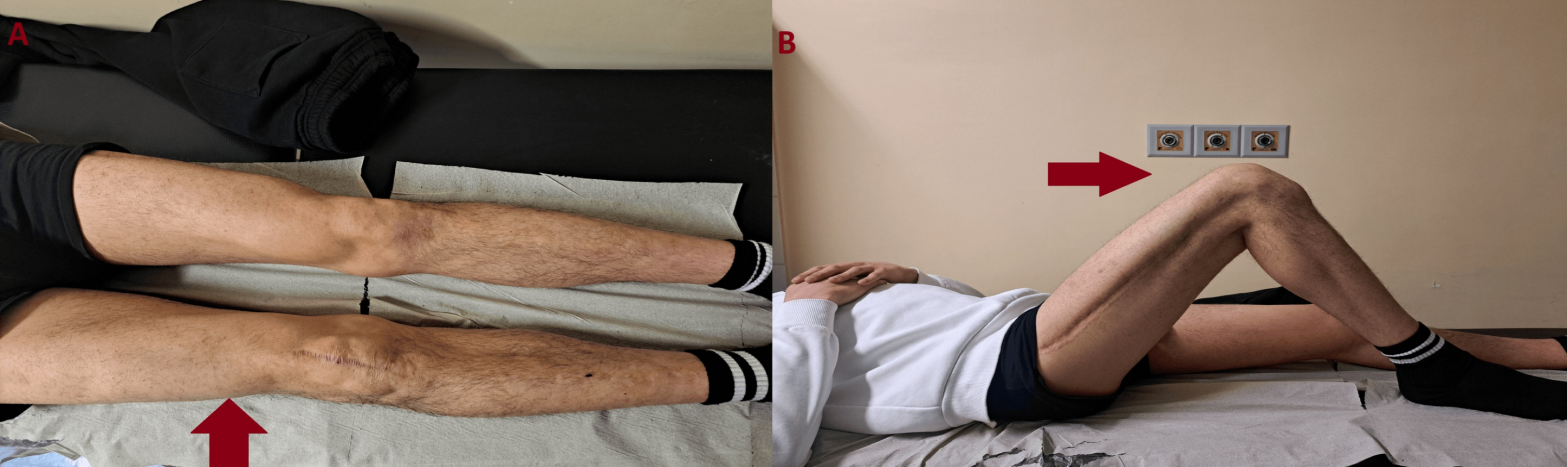
Obvious atrophy and limited flexion of the operated limb when compared with the healthy lower extremity A) Atrophy of the right quadriceps muscle. B) Limited flexion around 100 degrees in the operated limb

After these setbacks, the patient adhered to his surgeon's instructions. Nine months after the initial surgery, the femoral fracture had fully healed, with excellent knee extension. However, the patient exhibited limited knee flexion (100 degrees) and noticeable atrophy in his multi-injured right thigh (Figure 7).

## Discussion

Intramedullary nailing of femoral shaft fractures is a well-established method of indirect fixation, offering undeniable advantages over classic ORIF techniques. This has made it the preferred choice of treatment for the majority of orthopedic surgeons to stabilize shaft fractures of long bones. However, in some cases, intramedullary nailing may pose risks to the overall condition of the patient. Epidemiological studies indicate that approximately 30% of femoral shaft fractures occur in polytrauma patients, who may present with severe thoracic trauma and acute respiratory complications [4]. Fat embolism syndrome is another potential concern in polytrauma patients, particularly in the presence of a long bone fracture [5]. Recent studies investigating the impact of intramedullary nailing of long bone fractures on patients' respiratory function suggest that the increase in intramedullary pressure during nail insertion can raise the risk of fat embolism and pulmonary dysfunction [6]. Hence, although no established guidelines currently exist for the optimal surgical fixation method in these patients, many surgeons prefer alternative fixation techniques over nailing in cases of polytrauma patients with severe thoracic injuries and compromised cardiorespiratory function [7].

Most published studies on femoral shaft plating primarily focus on the pediatric population, as the widespread use of intramedullary nails has significantly reduced the frequency of open reduction and plate fixation in adults. Despite this trend, several studies suggest that techniques such as conventional single plate fixation, minimally invasive plate osteosynthesis (MIPO), and double plate fixation can serve as viable alternatives when nailing is contraindicated. These techniques have been shown to provide comparable final functional outcomes and support fast recovery [7-9]. However, while the overall results may be similar across these fixation methods, infection, malunion, and implant failure rates are reported to be higher with plate fixation compared to intramedullary nailing, according to the literature [10].

Another concern in our case is whether a revision fixation method, such as converting from ORIF with a plate to intramedullary nailing, yields similar functional outcomes and union rates compared to primary fixation. Studies specifically examining the functional outcomes of revision fixation methods for femoral diaphyseal fractures, in comparison to primary surgeries, are scarce. However, we can draw parallels to cases where patients have undergone a conversion from plate fixation to intramedullary nailing for femoral shaft non-unions. A systematic review by Somford et al. reported a union rate of 96% for failed plate constructs in femoral shaft non-unions, with excellent outcomes and recovery [11]. Our patient demonstrated excellent recovery following the second surgery before the patellar tendon rupture, with rapid imaging and clinical signs of union, as well as a great range of motion without any restrictions. It is undeniable though that a second operation, particularly when performed so soon after the primary procedure, significantly increases the risk of complications such as infection, delayed wound healing, and prolonged rehabilitation time [12].

Our brief review of the literature could not identify any studies linking femoral shaft fractures with late patellar tendon rupture as a postoperative complication. However, such injuries have been documented as concomitant injuries in high-energy traumatic events. A case report by Bek et al. [13] refers to a case of a neglected patellar tendon rupture in a patient who presented to the emergency department with an ipsilateral femoral shaft and tibial plateau fracture; the patellar tendon was reconstructed using semitendinosus and gracilis tendon grafts, resulting in a final range of motion of 5-110 degrees. Our patient demonstrated excellent knee extension but experienced restricted knee flexion, achieving a maximum of 100 degrees postoperatively. Similar outcomes are reported in recent literature, with authors noting high rates of patients returning to sports activities after primary patellar tendon repair, albeit with some degree of flexion loss when compared to the contralateral healthy knee [14,15]

Compliance with orthopedic surgeons' guidelines is crucial for achieving successful union and postoperative outcomes, particularly in cases of extremely comminuted lower extremity fractures where immediate weight-bearing might not be advised [16]. In a single-blinded study by Christopher et al., weight-bearing restrictions were tested using pressure-sensitive film embedded in short leg casts. The study revealed that a significant proportion of patients (approximately 27.5%) did not adhere to the non-weight-bearing guidelines. Interestingly, factors such as sex, age, language spoken, BMI, and the surgeon involved were not significant predictors of non-compliance. The only statistically significant factor associated with non-compliance was the time of year the cast was worn, with patients being more likely to stand on their injured limbs during warmer months than in colder months [17].

## Conclusions

This report highlights the importance of adhering to postoperative guidelines for ensuring successful outcomes after surgical management of femoral shaft fractures. While intramedullary nailing remains the gold standard, alternative fixation methods like plating are essential when contraindications exist. Revision to intramedullary nailing after failed plate fixation generally provides promising functional outcomes, but patient compliance with the surgeon’s guidelines is crucial to optimize recovery and prevent further complications. Careful surgical decision-making, particularly in polytrauma patients, and thorough follow-up care are essential for successful outcomes in femoral diaphyseal fractures.
